# Supplementary material for: Structure-evolution-designed amorphous oxides for dielectric energy storage
Source: Nat Commun. 2023 May 25;14:3031. doi: 10.1038/s41467-023-38847-1 (PMC10213053; doi:10.1038/s41467-023-38847-1)
Supplement: Supplementary file 1 — Supplementary Information [file 41467_2023_38847_MOESM1_ESM.pdf]

Supplementary Information for

Structure-evolution-designed amorphous oxides for dielectric energy storage

Yahui Yu,[1†] Qing Zhang,[2†] Zhiyu Xu,[1,3†] Weijie Zheng,[1] Jibo Xu,[1] Zhongnan Xi,[4] Lin Zhu,[4] Chunyan Ding,[1] Yanqiang Cao,[5] Chunyan Zheng,[1] Yalin Qin,[1] Shandong Li,[3] Aidong Li,[4] Di Wu,[4] Karin M. Rabe,[6] Xiaohui Liu,[2]* and Zheng Wen[1,3]*

[1]College of Physics, Qingdao University, Qingdao 266071, China

[2]School of Physics, Shandong University, Ji'nan 250100, China

[3]College of Electronics and Information, Qingdao University, Qingdao 266071, China

[4]National Laboratory of Solid-State Microstructures, Department of Materials Science and Engineering, Jiangsu Key Laboratory of Artificial Functional Materials and Collaborative Innovation Center for Advanced Materials, Nanjing University, Nanjing 210093, China

[5]Institute of Micro-nano Photonics and Quantum Manipulation, School of Science, Nanjing University of Science and Technology, Nanjing 210094, China

[6]Department of Physics and Astronomy, Rutgers University, Piscataway, New Jersey 08854, USA

This PDF file includes:

Supplementary Text 1

Figs. S1 to S15

Table S1

References (1-97)

Supplementary Text 1

As shown in the inset in Fig. 2, the monoclinic $P2_1/c$ phase gives the best fit with a R-factor of 0.0065 in the EXAFS spectrum of the BHO12-RT film, indicating a high-quality fitting. The fitting window is set to R = 1.0 ~ 4.0 Å, which is typical for the EXAFS analysis of amorphous structure because (i) R < 1.0 Å is meaningless since there is no interatomic distance shorter than 1.0 Å in actual crystals; (ii) the oscillation amplitude of EXAFS spectrum is too low to be fitted when R > 4.0 Å since the amorphous structure only has short-range ordering. The extracted structural parameters, including the interatomic distance ($R_{\text{Hf-O(or Hf)}}$), the coordination number ($N_{\text{Hf-O}}$), and the disorder (Debye-Waller) factor (σ^2), are listed in table below. The oscillation in R = 1.0 ~ 2.2 Å, *i.e.*, the first shell, can be attributed to single-scattering paths from the nearest-neighbor oxygen atoms at the $R_{\text{Hf-O}}$ of ~2.14 Å with the $N_{\text{Hf-O}}$ of ~6.35, while that in R = 2.0 ~ 3.5 Å is due to the single-scattering paths from Hf atoms at the $R_{\text{Hf-Hf}}$ of ~3.43 Å in the second shell. The σ^2 is 0.007 in the first shell. These results are in good agreement with the short-range structures reported previously in amorphous HfO_2 thin films.

However, the BHO12 exhibits two distinguished oscillations in 2.2 Å < R < 3.5 Å, which is obviously different from the unannealed BHO12-RT. Such a doublet feature makes the fit by only the $P2_1/c$ symmetry unavailable. The orthorhombic $Pca2_1$ symmetry shows a better fit and the best is obtained by combining the $P2_1/c$ and $Pca2_1$ phases, in which the single-scattering paths from the Hf atoms at the $R_{\text{Hf-Hf}}$ of ~3.42 Å (the $P2_1/c$) and the oxygen atoms at the $R_{\text{Hf-O}}$ of ~3.70 Å (the $Pca2_1$) contribute to the doublet oscillations of the second shell together. The R-factor is 0.0092 for the fitting window of R = 1.0 ~ 4.0 Å. In addition, the best fit also reveals that the oscillation in R = 1.0 ~ 2.2 Å is attributed to the single-scattering paths from oxygen atoms at the $R_{\text{Hf-O}}$

of 2.07 ~ 2.09 Å with the N_{Hf-O} of 6.75 ~ 6.81 in the first shell. Therefore, the BHO12 film exhibits a higher density compared to that prepared at room temperature since the Hf atoms are surrounded by more neighboring oxygen atoms with smaller coordination bond lengths. Also, due to the coexistence of $Pca2_1$ and $P2_1/c$ symmetries, the BHO12 has a large disorder factor of ~0.011.

	The first and second shell fits for EXAFS data of Hf L_{III} edge							
	$P2_1/c$				$Pca2_1$			
Sample ID	Scattering	R (Å)	N	σ^2 (Å^2)	Scattering	R (Å)	N	σ^2 (Å^2)
BHO12-RT	Hf-O	2.14±0.01	6.35±0.67	0.007±0.002				
	Hf-Hf	3.43±0.1	10.86±4.98	0.026±0.008				
BHO12	Hf-O	2.09±0.03	6.75±1.78	0.011±0.005	Hf-O	2.07±0.02	6.81±1.51	0.011±0.004
	Hf-Hf	3.42±0.07	8.76±12.02	0.029±0.025	Hf-O	3.70±0.05	5.43±4.5	0.004±0.013

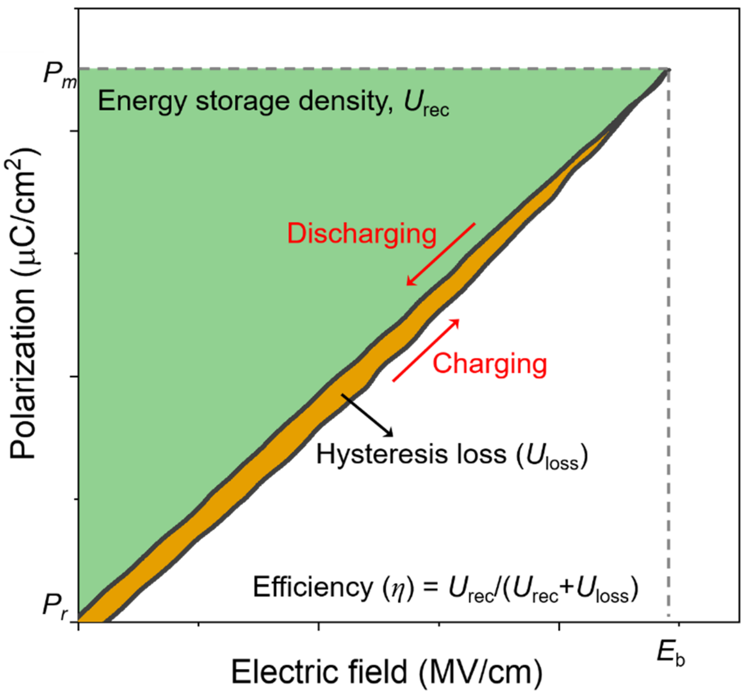

Figure S1. A sketch for the calculations of U_{rec} and η in the *P-E* hysteresis loop.

As shown in Fig. S1, the U_{rec} is calculated by $\int_{P_{\mathrm{r}}}^{P_{\mathrm{m}}} EdP$, indicated by the green area. The hysteresis area during a charging-discharging cycle is the U_{loss}. Then the η is obtained by $U_{\mathrm{rec}}/(U_{\mathrm{rec}}+U_{\mathrm{loss}})$.

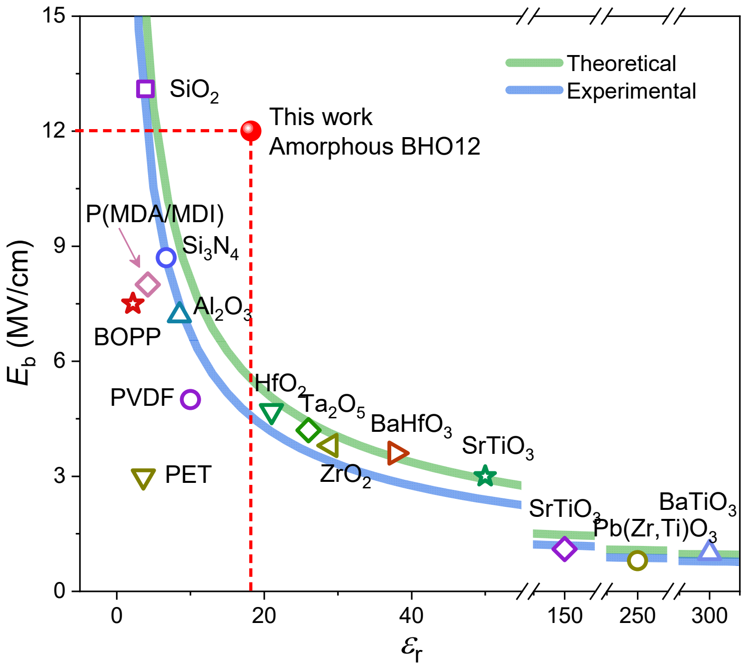

Figure S2. A comparison of the E_b and ε_r of the amorphous BHO12 to well-known dielectric materials for energy storage (BOPP: Biaxially oriented polypropylene; PVDF: Poly(vinylidene fluoride); PET: Poly(ethylene terephthalate); P(MDA/MDI): Aromatic polyurea (ploy(diaminodiphenylmethane dipheylmethane diisocyanate))).

Fig. S2 shows a comparison of the E_b and ε_r of the amorphous BHO12 to well-known high-κ, ferroelectric, and polymer materials for dielectric energy storage. In dielectric materials, the E_b is usually limited by ε_r, following $E_b = K * \varepsilon_r^{-\alpha}$, where K and α are constants. In the model by McPherson et al.,[1-3] the K and α are 35.3 and 0.64 for the theoretical (the thick green curve) and 29.9 and 0.65 for the experimental (the thick blue curve), respectively. One can find that these dielectric materials are following this permittivity limitation. However, the breakdown strength of amorphous BHO12 is much higher than the upper-limit of its permittivity, overcoming the negative correlation between E_b and ε_r. In ref.1 of the main text, a comprehensive review of capacitive energy storage, the authors have also fitted the experimental E_b and ε_r of dielectric materials using $E_b = K * \varepsilon_r^{-\alpha}$ and extracted the K and α, which are 25.09 and 0.559, respectively, very close to the model used in Fig. S2.

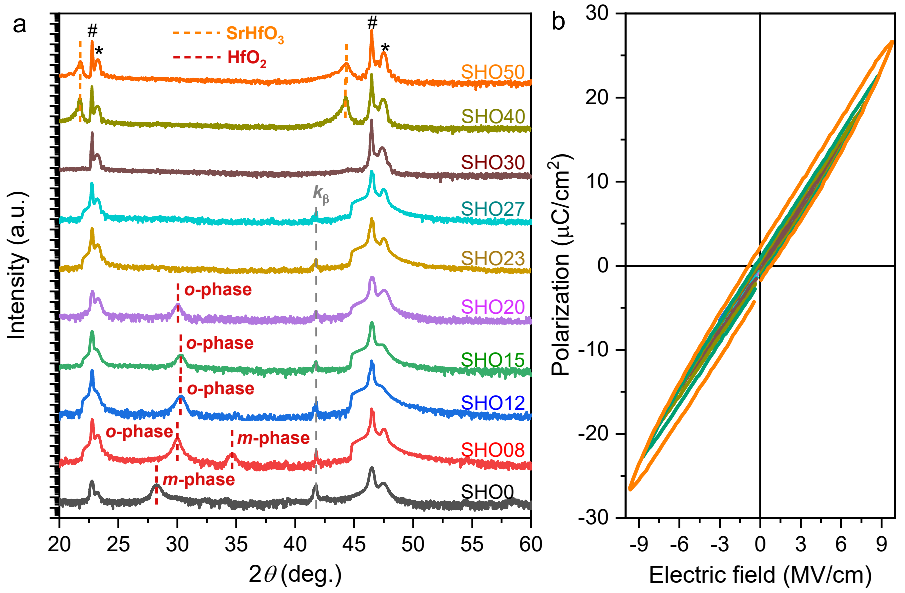

Figure S3. a, XRD patterns of the Sr-substituted HfO_2 thin films with increasing substitution concentration from 0 to 50 %. **b**, *P-E* hysteresis loops of the amorphous Pt/SHO30/LSMO capacitor at 10 kHz.

As shown in Fig. S3a, the amorphous Sr-substituted HfO_2 (SHO*x*) thin films appear at the concentration of 23% ~ 30%, which is narrower than that of the BHO, in agreement with that observed in the first-principles calculation. For the amorphous Pt/SHO30/LSMO capacitor, the E_b is above 10 MV/cm (Fig. S3b), which is also much higher than that reported in the amorphous and crystalline HfO_2-based capacitors. The U_{rec} and η calculated by the *P-E* loop is 117 J/cm^3 and 76.8% at E = 10 MV/cm.

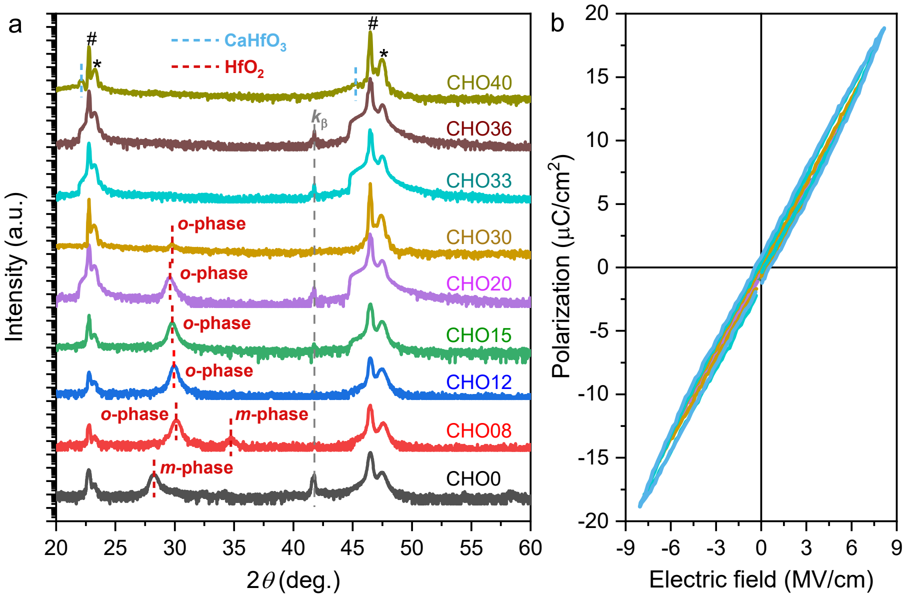

Figure S4. **a**, XRD patterns of the Ca-substituted HfO_2 thin films with increasing substitution concentration from 0 to 50 %. **b**, *P-E* hysteresis loops of the amorphous Pt/CHO33/LSMO capacitor at 10 kHz.

As shown in Fig. S4a, the amorphous Ca-substituted HfO_2 (CHO*x*) thin films appear at the Ca concentration of 33% ~ 36%, which is narrower than that of the SHO and BHO, in agreement with that observed in the first-principles calculation. For the amorphous Pt/CHO33/LSMO capacitor, the E_b is above 8.0 MV/cm (Fig. S4b), which is also much higher than that reported in the amorphous and crystalline HfO_2-based capacitors. The U_{rec} and η calculated by the *P-E* loop is 72 J/cm^3 and 82.4% at E = 8.3 MV/cm.

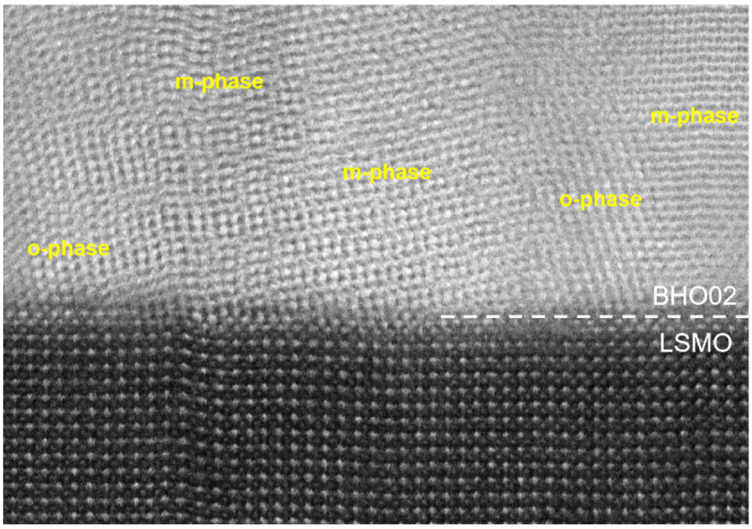

Figure S5. HAADF image of the BHO02/LSMO/STO heterostructure to show the coexistence of *m*- and *o*-phase.

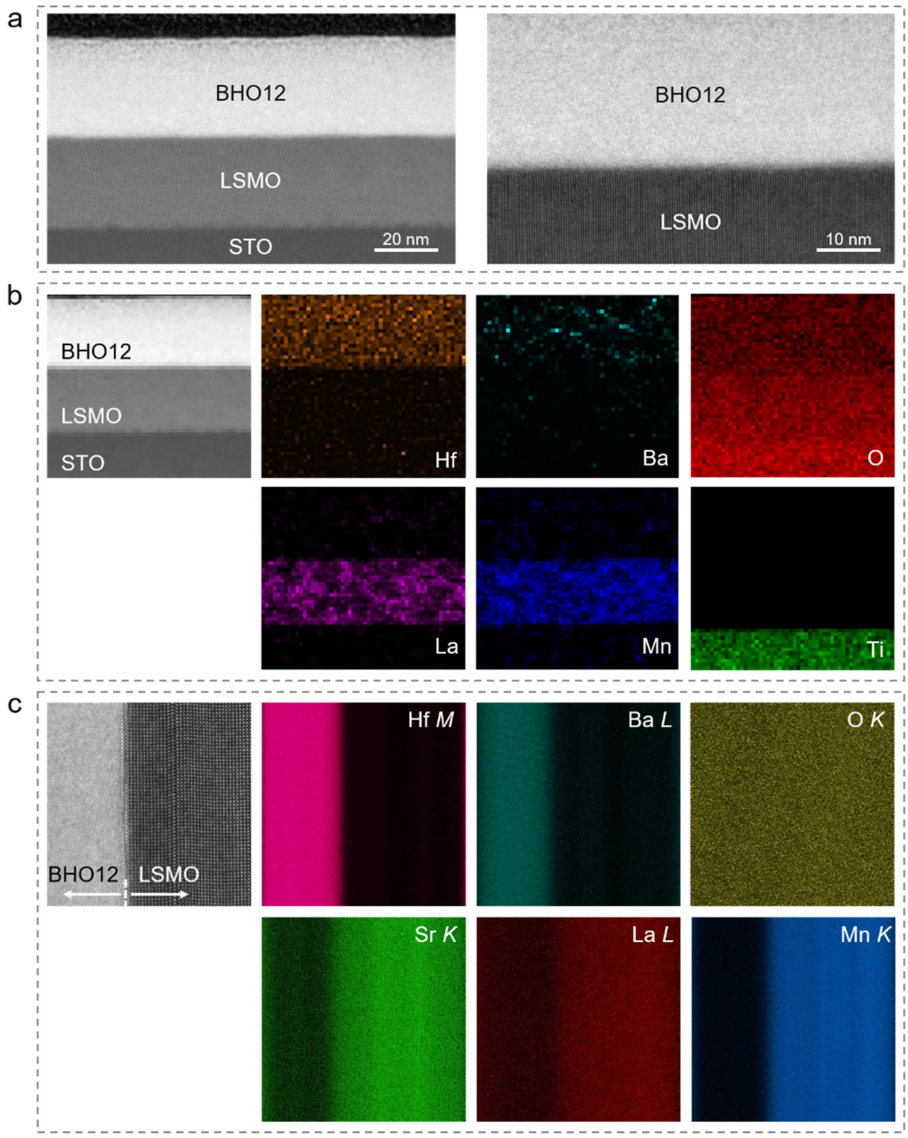

Figure S6. STEM characterizations of the BHO12/LSMO/STO heterostructure in a large scale to show the uniformity in amorphous structure (**a**) and element distributions (**b**, **c**). **b**, the EELS mappings for Hf, Ba, O, La, Mn, and Ti elements, respectively. **c**, the EDS (energy dispersive spectra) mappings for Hf, Ba, O, Sr, La, and Mn elements, respectively.

In Fig. S6a, one can find that the amorphous structure is uniform over a large area with the scale bar of 20 nm. Combined with the EELS (Fig. S6b) and EDS (Fig. S6c) mapping, one can find the element distributions are also uniform.

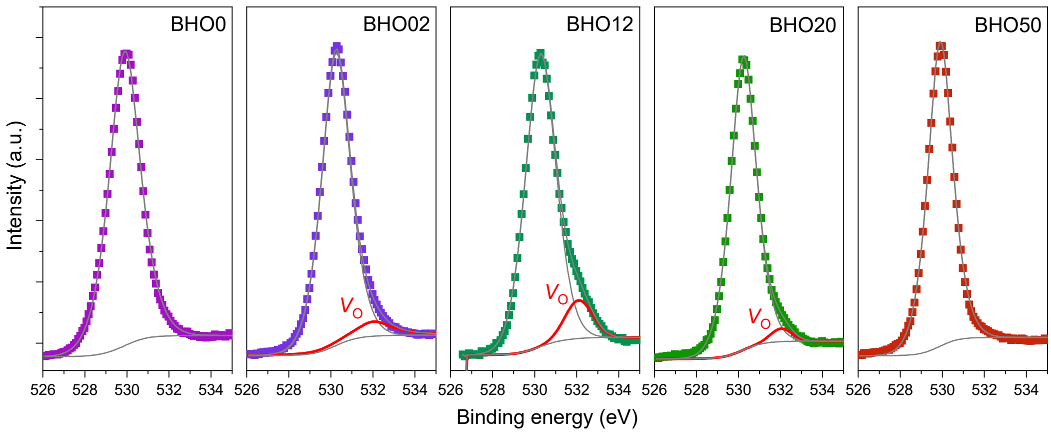

Figure S7. XPS spectra of O 1*s* core level for the BHO0, BHO02, BHO12, BHO20, and BHO50 thin films, respectively.

Fig. S7 shows XPS spectra of O 1*s* core level for the BHO thin films etched by Ar ions for 30 s to remove the adsorbed oxygen. In the HfO_2-based films the peaks of V_O are in general present at the binding energy of ~532 eV, higher than that of the lattice oxygen ions.[4,5] In Fig. S7, one can find that the V_Os in the BHO0 and BHO50, that is the HfO_2 and $BaHfO_3$, are negligible owing to the post-annealing in flowing O_2. With the Ba substitution, the V_Os appear in BHO02, BHO12, and BHO20, in agreement with the first-principle calculation. In addition, the relative concentration of V_Os increases from the BHO02 to the BHO12 and then decreases in the BHO20.

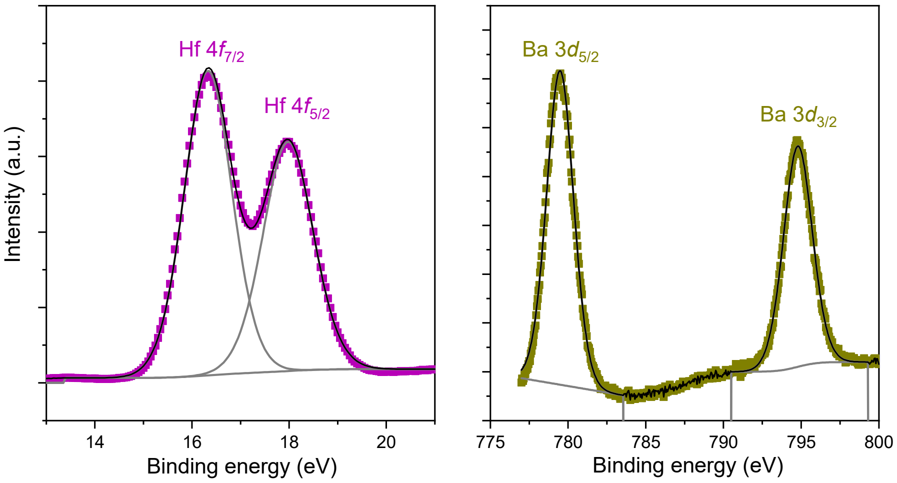

Figure S8. XPS spectra of Hf 4*f* and Ba 3*d* core levels of the BHO12 thin film.

In the XPS spectrum of Hf 4*f* core level, the peaks at 16.3 and 18.0 eV are Hf $4f_{7/2}$ and Hf $4f_{5/2}$, which are attributed to the Hf^{4+} of Hf-O bond. In the XPS spectrum of Ba 3*d* core level, the Ba $3d_{5/2}$ and $3d_{3/2}$ appear at 779.5 and 794.8 eV, respectively, separated by 15.3 eV, which correspond to the Ba^{2+} in Ba-O bond. There are no metallic Hf and Ba observed in the amorphous BHO12 thin film.

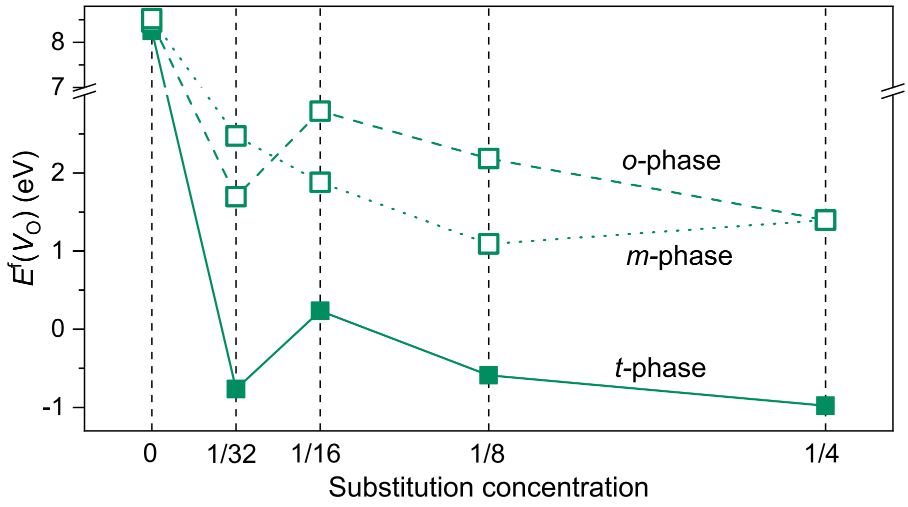

Figure S9. The $E^f(V_O)$ at the first nearest-neighbor site as a function of Ba^{2+} substitution concentration in different phases.

In the main text, the Ba substitution induces strong effect on the oxygen instability in the *t*-phase HfO_2, which is the high-temperature phase considering that the amorphous state is formed in the crystallizing process of the Ba-Hf-O system. However, the amorphous HfO_2 films reported in literature are in general formed by low-temperature deposition. In this case, the HfO_2 is in a low symmetry, like the *m*- and *o*-phases. We therefore calculate the $E^f(V_O)$ in the *m*- and *o*-phase HfO_2 lattices, for comparison. As shown by the dashed and dotted curves in Fig. S9, the $E^f(V_O)$ are always positive with increasing Ba concentration from 1/32 to 1/4, indicating that the Ba substitution yields weak effect on the oxygen instability in the *m*- and *o*-phases. These results also suggest that the low-temperature stacking of *A*/Hf and O atoms cannot generate the amorphous structure like the BHO films in the main text, in agreement with the short-range structures observed in the EXAFS spectra.

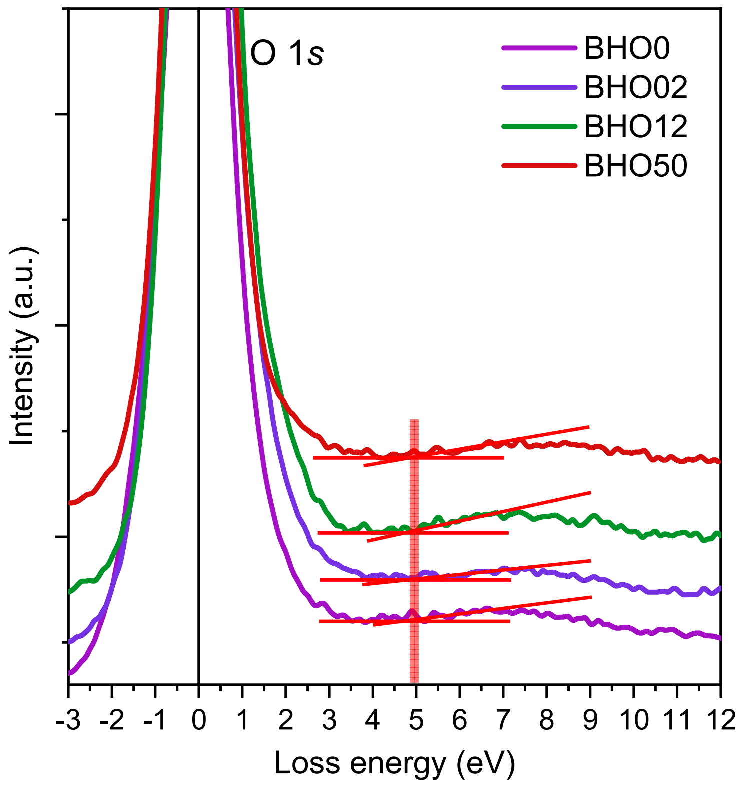

Figure S10. O 1*s* energy loss spectra of the BHO0, BHO02, BHO12, and BHO50 thin films.

Considering that the BHO thin films are grown on the LSMO/STO and the bandgap of BHO should be higher than that of the STO substrate, we adopted the O 1*s* energy loss spectra to estimate their bandgaps, which have been reported frequently in the band structure studies of high-κ thin films.[6,7] As shown in Fig. S10, the bandgaps of the representative BHO thin films are about 5.0 eV, regardless of crystalline or not. These results are reasonable since the bandgaps of HfO_2 and $BaHfO_3$ are about 4.0 ~ 6.0 eV, as reported previously.[1,8,9]

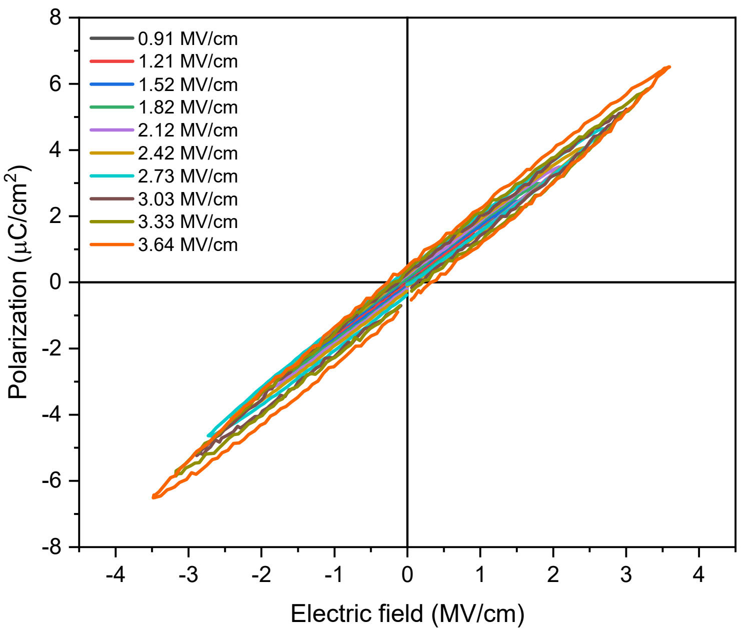

Figure S11. *P-E* hysteresis loops of the Pt/BHO12-RT/LSMO capacitor measured at 10 kHz

As shown in Fig. S11, the capacitor based on the BHO12-RT thin film exhibits an E_b of ~3.64 MV/cm, which is comparable with that reported in the amorphous HfO_2 thin films.[10]

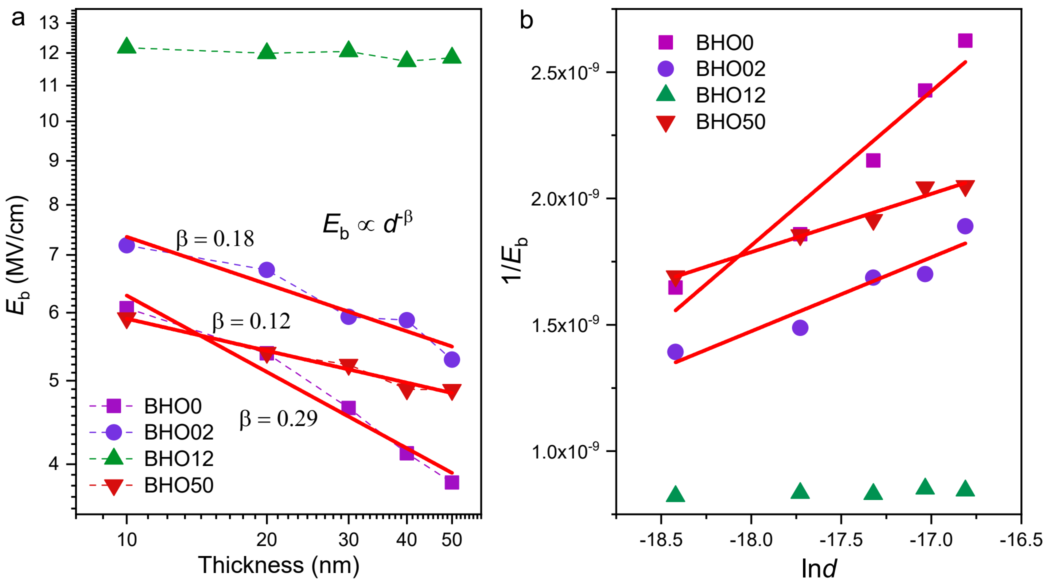

Figure S12. a, The E_b of BHO0, BHO02, BHO12, and BHO50 capacitors as a function of thin-film thickness. **b**, Replotted the thickness-dependent breakdown strength in $1/E_b$ vs. ln*d*.

In the avalanche mechanism, the E_b usually exhibits a thickness-dependence character, following an empirical formula $E_b \propto d^{-\beta}$, in which β is a constant and d is thickness. In Fig. S12a, one can find that the breakdown strengths of the crystalline BHO0, BHO02, and BHO50 capacitors are decreasing with increasing d from 10 to 50 nm, exhibiting the β of 0.29, 0.18, and 0.12, respectively. However, the E_b of amorphous BHO12 capacitor is almost independent with the thickness. The thickness-dependent E_b is further analyzed by the 40-generation-electron theory, which obeys

$$E_b = {}^{k}\!\big/\ln\left(\frac{d}{d_0}\right) \qquad \text{(S1)}$$

where $d_0 = 40/\alpha_0$ (α_0 is the ionization coefficient) and k is a parameter with the same unit of electric field. By linear fitting the $1/E_b$ vs. ln$d$ plots, the d_0 and k can be extracted, which are 1.27 nm and 16 MV/cm, 0.11 nm and 34 MV/cm, and 0.008 nm and 43 MV/cm for the BHO0, BHO02, and BHO50 capacitors, respectively.

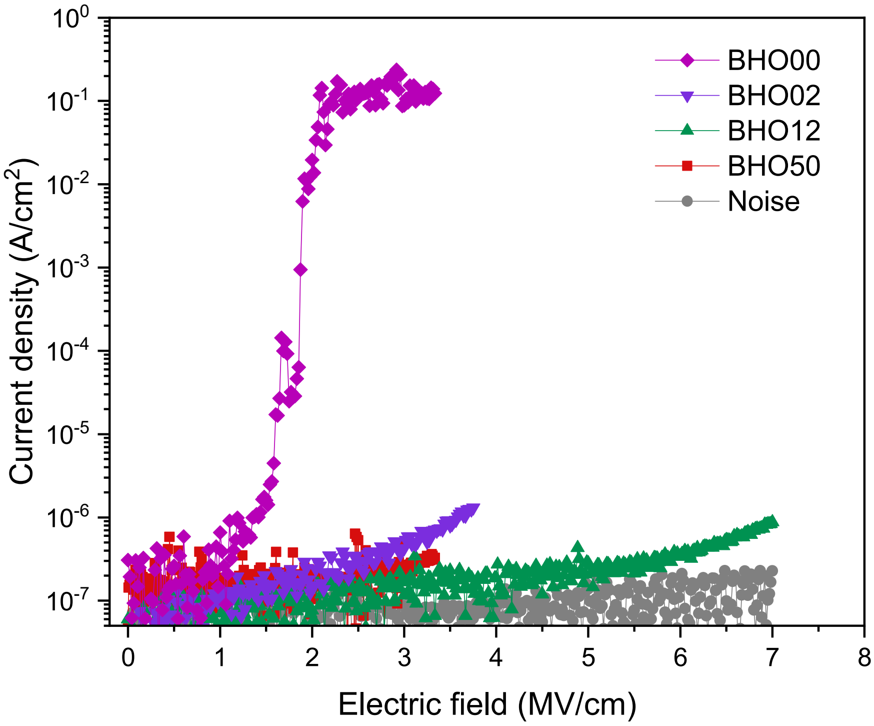

Figure S13. Current density-electric field curves of the BHO0, BHO02, BHO12, and BHO50 thin-film capacitors. Here, the noise of our facilities is also shown for comparison.

As shown in Fig. S13, the BHO12 capacitor exhibits the lowest leakage current density, which is even close to the current noise (~1.0 pA) of our facilities. At E = 7.0 MV/cm, the current density is still less than 1×10^{-6} A/cm^2.

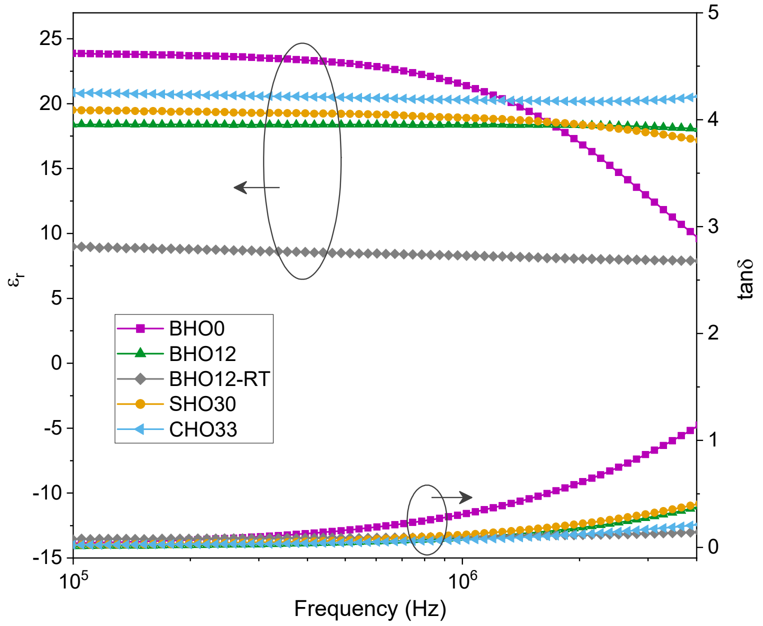

Figure S14. ε_r and dielectric loss (tan δ) of the BHO0, the amorphous BHO12, SHO30, and CHO33, as well as the unannealed BHO12-RT, as a function of frequency.

As shown in Fig. S14, the ε_r of amorphous BHO12, SHO30, and CHO33 thin-film capacitors are about 18 ~ 21 in the frequency range of $1\times10^5 \sim 4\times10^6$ Hz, which is much larger than that of the unannealed BHO12-RT counterpart and even larger than the crystalline BHO0 (i.e., the undoped HfO_2) capacitor at high frequency. In addition, these amorphous hafnium-based oxides also exhibit very low dielectric loss.

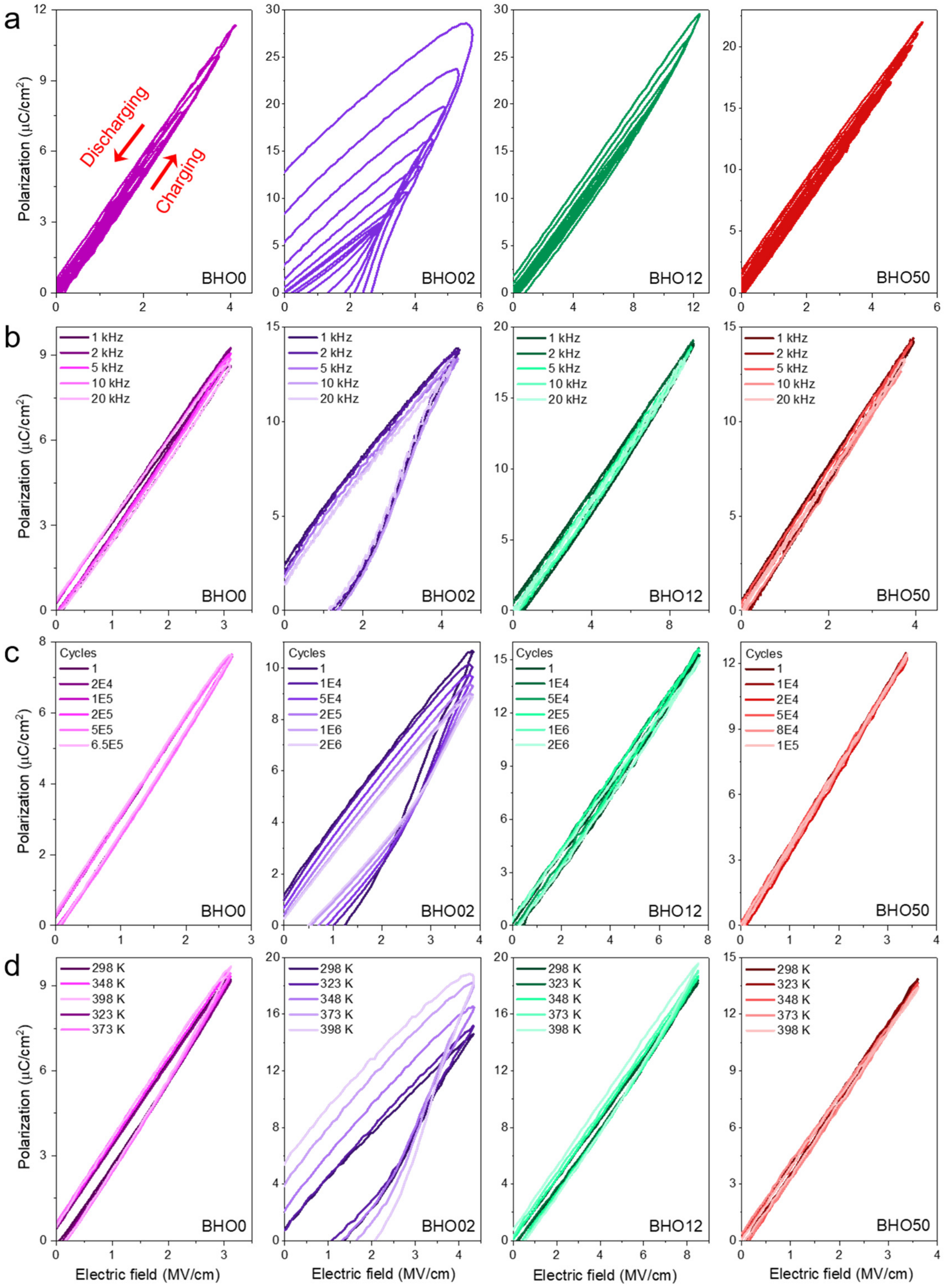

Figure S15. *P-E* hysteresis loops of the BHO0, BHO02, BHO12, and BHO50 capacitors as functions of applied *E* (**a**), measurement frequency (**b**), charging/discharging cycling number (**c**), and temperature (**d**), respectively.

Fig. S15a-d are the corresponding *P-E* hysteresis loops of the data plotted in Fig.

4a-d, respectively. Here, we give a discussion on the BHO02 capacitor since it shows a large change in the η with the measurement conditions due to the ferroelectric behaviors. In Fig. S15a, the ferroelectric hysteresis becomes stronger with increasing E, resulting in the decrease of η (Fig. 4a). In Fig. S15c, the BHO02 capacitor shows a polarization fatigue phenomenon with increasing cycling number, in which the remanent polarization is decreased and thus the hysteresis is suppressed. It results in the increase of η with increasing cycling number in Fig. 4c. In Fig. S15d, the BHO02 exhibits a weak-up effect of ferroelectric polarization, resulting in the increase of hysteresis and the decrease of η with increasing temperature (Fig. 4d).

Table S1. The dielectric energy storage properties, including device structure, ε_r, E_b, U_{rec}, and η, for representative thin-film capacitors fabricated by different material systems,[11-97] in which the symbols for dielectric capacitors plotted in Fig. 4e are indicated.

Type	Dielectric films	Film thickness	ε_r	E_b (MV cm^{-1})	U_{rec} (J cm^{-3})	η (%)	Ref.
High-κ binary oxides	**BHO12**<br>**SHO30**<br>**CHO33**	30 nm	18~21	**12**<br>**10**<br>**8.3**	155<br>116.86<br>71.83	87<br>76.8<br>82.4	This work
	$Ta_2O_5/Hf_{0.5}Zr_{0.5}O_2$ (◆)	25 nm	-	**7.2**	109	94.4	[11]
	Al:HfO_2 (▼)	6.5 nm	-	**6.5**	63.7	64.2	[12]
	$Hf_{0.5}Zr_{0.5}O_2$/ $Hf_{0.25}Zr_{0.75}O_2$ (★)	10 nm	-	**6**	71.95	57.8	[13]
	$Hf_{0.67}Zr_{0.33}O_2/Al_2O_3$ /$Hf_{0.67}Zr_{0.33}O_2$ (▲)	10 nm	-	**6**	70	50	[14]
	ZrO_2 (●)	470 nm	26	**5.8**	75.4	88	[15]
	$TiO_2/ZrO_2/TiO_2$ (■)	8.7 nm	-	**5.5**	94	80	[16]

	Al:HfO_2 (▲)	50 nm	26	**5**	63	85	[17]
	$Hf_{0.5}Zr_{0.5}O_2$ (▲)	7.1 nm	40	**5**	55	57	[18]
	ZrO_2/Al:HfO_2 (▼)	7 nm	-	**5**	54.3	51.3	[19]
	Si:HfO_2 (●)	10 nm	-	**4.5**	61.2	65	[20]
	Si:$Hf_{0.5}Zr_{0.5}O_2$ (●)	10 nm	-	**4.5**	50	80	[21]
	$Hf_{0.3}Zr_{0.7}O_2$ (●)	9.2 nm	42~44	**4.35**	45	50	[22]
	HfO_2 (★)	63 nm	26	**4.25**	21.3	75	[23]
	La:$Hf_{0.5}Zr_{0.5}O_2$ (◆)	10 nm	45	**4**	50	70	[24]
	HfO_2/ZrO_2 (⬢)	8.8 nm	-	**4.0**	49.9	61.87	[25]
	Al:HfO_2 (●)	10 nm	56	**4**	40.58	50	[26]
	Si:HfO_2 (■)	9 nm	34	**3.33**	40	80	[27]
	Al:HfO_2 (■)	50 nm	32.28	**3**	18.17	51.79	[28]

		$Hf_{0.3}Zr_{0.7}O_2$ (■)	6 nm	-	**2.5**	35.4	69.3	[29]
Perovskite oxides	Paraelectrics	$0.90BaTiO_3$-$0.08Bi(Ni_{0.5}Zr_{0.5})O_3$-$0.02BiFeO_3$ (⬦)	120 nm	-	**8.3**	114.3	87.0	[30]
		$SrTiO_3$ (△)	610 nm	-	**6.6**	307	89	[31]
		$(Bi_{3.25}La_{0.75})(Ti_{1.8}Zr_{0.4}Hf_{0.4}Sn_{0.4})O_{12}$ (⊖)	620 nm	-	**6.35**	182	78	[32]
		$Bi_{1.5}Zn_{0.9}Ta_{1.5}O_{6.9}$ (⊟)	150 nm	55	**6.1**	60.7	-	[33]
		$Bi_{1.5}Zn_{0.9}Nb_{1.35}Ta_{0.15}O_{6.9}$ (⬠)	150 nm	122	**5.5**	66.9	-	[33]
		$Bi_{1.5}Zn_{0.9}Nb_{1.5}O_{6.9}$ (☆)	150 nm	145	**5.1**	60.8	-	[33]
		$Ba_{0.53}Sr_{0.47}TiO_3$ (▽)	170 nm	-	**4.82**	51.2	54.3	[34]
		3 mol% Mn-doped $0.94BaTiO_3$-$0.06Bi(Zn_{1/2}Zr_{1/2})O_3$ (⬡)	175 nm	-	**4.75**	85	84	[35]
		1 mol% Mn-doped $SrTiO_3$ (▷)	140 nm	43	**4.54**	53.9	77.2	[36]
		$0.94BaTiO_3$-$0.06Bi(Zn_{1/2}Zr_{1/2})O_3$ (◁)	175 nm	-	**4.50**	51.6	62.2	[35]
		$Bi_2Zn_{2/3}Nb_{4/3}O_7$ (◇)	150 nm	182	**3.81**	63.5	61.13	[37]

		$Ba_{0.53}Sr_{0.47}TiO_3$ (▽)	170 nm	-	**3.4**	36.1	68.1	[38]
		$Bi_{1.5}MgNb_{1.5}O_7$-$Bi_2Mg_{2/3}Nb_{4/3}O_7$ (△)	100 nm	158	**2.91**	40.59	61.67	[39]
		3 mol% Mn-doped $Ba_{0.4}Sr_{0.6}TiO_3$ (○)	100 nm	-	**1.63**	8.48	42.4	[40]
		$Ba_{0.7}Sr_{0.3}Fe_{0.008}Ti_{0.092}O_3$ (□)	100 nm	300	**0.4**	7.6	65	[41]
		$BaZr_{0.35}Ti_{0.65}O_3$/1 mol% SiO_2-doped $BaZr_{0.35}Ti_{0.65}O_3$/$BaZr_{0.35}Ti_{0.65}O_3$ (⊕)	400 nm	158	**9.39**	130.1	73.8	[42]
		$BaTiO_3$ (⊞)	160 nm	-	**8.75**	242	76	[43]
		$BaTiO_3$ (⬠)	200 nm	110	**7.0**	130	76	[44]
	Ferroelectrics	$PbZr_{0.52}Ti_{0.48}O_3$/Al_2O_3/$PbZr_{0.52}Ti_{0.48}O_3$ (☆)	330 nm	43	**5.71**	63.7	81.3	[45]
		$Ba(Zr_{0.2}Ti_{0.8})O_3$ (⬡)	350 nm	-	**5.7**	166	78	[46]
		$BaZr_{0.25}Ti_{0.75}O_3$ (▷)	180 nm	-	**5.22**	60.8	87.8	[47]
		$Bi(Mg_{0.5}Ti_{0.75})O_3$ (◁)	120 nm	-	**5.0**	126	-	[48]

		$Sr_{0.99}(Na_{0.5}Bi_{0.5})_{0.01}(Ti_{0.99}Mn_{0.01})O_3$ (⬙)	200 nm	158.6	**4.91**	53	48.8	[49]
		$BiAlO_3$ (▽)	140 nm	-	**3.54**	31.1	82.2	[50]
		$Bi_{0.5}Na_{0.5}TiO_3/Al_2O_3/Bi_{0.5}Na_{0.5}TiO_3$ (△)	338 nm	228	**3.08**	19.9	49.8	[51]
		0.5 mol% $0.91Bi_{3.15}Nd_{0.85}Ti_{2.8}Zr_{0.2}O_{12}$-$0.09BiFeO_3$ (⊖)	350 nm	-	**3.01**	124	81.9	[52]
		$0.4SrTiO_3$-$0.6Bi_{3.25}La_{0.75}Ti_3O_{12}$ (⊟)	300 nm	244	**3.0**	44.7	87.4	[53]
		$Bi_{3.25}La_{0.75}Ti_3O_{12}/BiFeO_3/Bi_{3.25}La_{0.75}Ti_3O_{12}$ (⬠)	320 nm	298	**2.75**	65.5	74.2	[54]
		$BiFe_{0.93}Mn_{0.07}O_3/Ba_2Bi_4Ti_5O_{18}$ (☆)	375 nm	-	**2.5**	52.6	75.9	[55]
		$Bi_{3.25}La_{0.75}Ti_3O_{12}$ (⬡)	480 nm	-	**2.4**	44.7	78.4	[56]
		$Ba_2Bi_4Ti_5O_{18}$ (▷)	410 nm	-	**2.36**	41.2	79.1	[55]
		$SrTiO_3/BiFeO_3/SrTiO_3$ (◁)	170 nm	274	**2.06**	40.69	44.7	[57]
		$0.95(Na_{0.5}Bi_{0.5})TiO_3$-$0.05SrTiO_3$ (◇)	1.5 μm	964	**1.97**	36.1	40.8	[58]

		$BiFeO_3/BaTiO_3$ (▽)	980 nm	380	**1.94**	71	61	[59]
		$BiMg_{0.56}Ti_{0.5}O_3$ (△)	-	-	**1.80**	44.1	80	[60]
		$Pb(Zr_{0.52}Ti_{0.48})O_3$ (○)	1 μm	1260	**1.0**	10.3	62.4	[61]
		$BiFeO_3$ (□)	40 nm	-	**0.5**	3.2	-	[54]
	Anti-ferroelectrics	$Pb_{0.5}Sr_{0.5}HfO_3$ (⊟)	75 nm	52	**5.12**	77	97	[62]
		$Pb_{0.88}Ca_{0.12}ZrO_3$ (⬠)	300 nm	58	**4.99**	91.3	85.3	[63]
		$PbZrO_3/Pb_{0.9}La_{0.1}Zr_{0.52}Ti_{0.48}O_3$ multilayer (☆)	500 nm	-	**4.4**	128.4	81.2	[64]
		$Pb_{0.96}La_{0.04}Zr_{0.98}Ti_{0.02}O_3$ (⬡)	690 nm	350	**4.3**	61	33	[65]
		$Pb_{0.97}La_{0.02}(Zr_{0.66}Sn_{0.23}Ti_{0.11})O_3$ (▷)	650 nm	-	**4.0**	46.3	84	[66]
		$(Pb_{0.97}La_{0.02})(Zr_{0.91}Sn_{0.04}Ti_{0.05})O_3$ (◁)	1.8 μm	525	**3.71**	56	-	[67]
		$PbHfO_3$ (◇)	330 nm	-	**3**	25	73	[68]

		$Pb_{0.82}La_{0.12}Zr_{0.85}Ti_{0.15}O_3$ (▽)	1 μm	433	**2.14**	38	71	[69]
		$Pb_{0.85}La_{0.1}ZrO_3$ (△)	450 nm	-	**1.4**	23.1	73	[70]
		$Pb_{0.97}Y_{0.02}[(Zr_{0.6}Sn_{0.4})_{0.925}Ti_{0.075}]O_3$ (○)	500 nm	-	**1.3**	21.0	91.9	[71]
		$PbHfO_3$ (□)	200 nm	-	**1.25**	21.41	70	[72]
	Relaxor ferroelectrics	$BaZr_{0.35}Ti_{0.65}O_3$ (✵)	295 nm	-	**8.7**	100.8	78.0	[73]
		$0.85BaTiO_3$-$0.15Bi(Mg_{0.5}Zr_{0.5})O_3$ (◈)	300 nm	-	**8.17**	99.34	75.65	[74]
		$0.68Pb(Mg_{1/3}Nb_{2/3})O_3$-$0.32PbTiO_3$ (✳)	175 nm	-	**5.92**	133.3	75	[75]
		30 mol% Sm-doped $0.3BiFeO_3$-$0.7BaTiO_3$ (+)	650 nm	-	**5.2**	152	77	[76]
		$0.88Ba_{0.55}Sr_{0.45}TiO_3$-$0.12BiMg_{2/3}Nb_{1/3}O_3$ (△)	400 nm	225	**5.0**	86	73	[77]
		$0.25BiFeO_3$-$0.30BaTiO_3$-$0.45SrTiO_3$ (⊕)	450 nm	-	**4.9**	112	80	[78]
		$(0.4BiFeO_3$-$0.6SrTiO_3)/Ba_{0.5}Sr_{0.5}TiO_3$ multilayer (⊞)	410 nm	471	**4.76**	98	80	[79]

		$Ba_{0.7}Ca_{0.3}TiO_3/BaZr_{0.2}Ti_{0.8}O_3$ multilayer (⬠)	100 nm	-	**4.5**	52.4	72.3	[80]
		$0.25BiFeO_3$-$0.75SrTiO_3$ (☆)	500 nm	-	**4.46**	70	68	[81]
		$0.30BiFeO_3$-$0.35BaTiO_3$-$0.35SrTiO_3$ (⊖)	500 nm	-	**4.0**	79	78	[82]
		$0.4BiFeO_3$-$0.6SrTiO_3$ (▷)	500 nm	-	**3.85**	70.3	70	[81]
		Sm-doped $BaZr_{0.2}Ti_{0.8}O_3$ (◁)	200 nm	-	**3.68**	40.42	85.03	[83]
		$Pb_{0.9}La_{0.1}(Zr_{0.52}Ti_{0.48})O_3$ (◇)	1 μm	-	**3.6**	68.2	80.4	[61]
		0.5 mol% Mn-doped $0.4BiFeO_3$-$0.6SrTiO_3$ (▽)	500 nm	-	**3.6**	51	64	[84]
		(100) $BaZr_{0.3}Ti_{0.7}O_3$ (△)	400 nm	3446	**3.0**	156	72.8	[85]
		$Pb_{0.9}La_{0.1}(Zr_{0.52}Ti_{0.48})O_3$ (⊖)	1.2 μm	-	**3.0**	40.9	80.2	[86]
		$BaZr_{0.2}Ti_{0.8}O_3$ (⊟)	90 nm	-	**3.0**	30.4	81.7	[87]
		0.01 mol% Mn-doped $0.55Na_{0.5}Bi_{0.5}TiO_3$-$0.45Sr_{0.2}Bi_{0.7}TiO_3$ (⬠)	360 nm	-	**2.86**	30.5	65	[88]

		La-doped 0.9$Na_{0.5}Bi_{0.5}TiO_3$-0.1$BiFeO_3$ (☆)	330 nm	-	**2.7**	52.4	60.3	[89]
		(0.7$Na_{0.5}Bi_{0.5}TiO_3$-0.3$SrTiO_3$)/(0.6$SrTiO_3$-0.4$Na_{0.5}Bi_{0.5}TiO_3$) multilayer (⬡)	190 nm	-	**2.61**	60	51	[90]
		0.6$PbTiO_3$-0.4$Bi(Mg_{0.5}Zr_{0.5})O_3$ (▷)	500 nm	-	**2.6**	32.3	51.4	[91]
		$Pb_{0.9}La_{0.1}(Zr_{0.52}Ti_{0.48})O_3$/$Pb(Zr_{0.52}Ti_{0.48})_{0.99}Nb_{0.01}O_3$ (◁)	200 nm	-	**2.45**	43.5	84.1	[92]
		$BaBi_4Ti_4O_{15}$ (◇)	450 nm	314	**2.06**	44.3	87.1	[93]
		0.9$Na_{0.5}Bi_{0.5}TiO_3$-0.1$BiFeO_3$ (▽)	500 nm	491	**2.0**	38.5	52.0	[94]
		0.65$Pb(Mg_{1/3}Nb_{2/3})O_3$-0.35$PbTiO_3$ (△)	2 μm	-	**2.0**	35	70	[95]
		Mn-doped $Pb_{0.97}La_{0.02}(Zr_{0.905}Sn_{0.015}Ti_{0.08})O_3$ (○)	350 nm	-	**2.0**	31.2	58	[96]
		$Na_{0.5}Bi_{0.5}TiO_3$ (□)	400 nm	405	**1.25**	23.3	61.6	[97]

References

1. Song, D. et al. High-*k* perovskite gate oxide for modulation beyond 10^{14} cm^{-2}. *Sci. Adv.* **8**, eabm3962 (2022).

2. McPherson, J., Kim, J., Shanware, A., Mogul, H. & Rodriguez, J. Proposed universal relationship between dielectric breakdown and dielectric constant. *IEDM Technical Digest* 633-636 (2002).

3. McPherson, J. W., Kim, J., Shanware, A., Mogul, H. & Rodriguez, J. Trends in the ultimate breakdown strength of high dielectric-constant materials. *IEEE Tran. Electr. Dev.* **50**, 1771-1778 (2003).

4. Luo, X. G. et al. Investigation of HfO_2 thin films on Si by X-ray photoelectron spectroscopy, Rutherford backscattering, grazing Incidence X-ray diffraction and variable angle spectroscopic ellipsometry. *Crystals* **8**, 248 (2018).

5. Bon, C. Y. et al. Enhanced electrical properties of Nb-doped *a*-HfO_2 dielectric films for MIM capacitors. *AIP Advances* **10**, 115117 (2020).

6. Yu, H. Y., Li, M. F. & Kwong, D. L. ALD $(HfO_2)_x(Al_2O_3)_{1-x}$ high-*k* gate dielectrics for advanced MOS devices application. *Thin Solid Films* **462**, 110-113 (2004).

7. Gong, Y.-P. et al. Impact of the Al/Hf ratio on the electrical properties and band alignments of atomic-layer-deposited HfO_2/Al_2O_3 on S-passivated GaAs substrates. *Semicond. Sci. Technol.* **25**, 055012 (2010).

8. Yim, K. et al. Novel high-*k* dielectrics for next-generation electronic devices screened by automated ab initio calculations. *NPG Asia Mater.* **7**, e190 (2015).

9. Kim, Y. M. et al. High-*k* perovskite gate oxide $BaHfO_3$. *APL Mater.* **5**, 016104 (2017).

10. Reklaitis, I. et al. A comparative study on atomic layer deposited oxide film morphology and their electrical breakdown. *Surf. Coat. Tech.* **399**, 126123 (2020).

11. Hoffmann, M. et al. Negative capacitance for electrostatic supercapacitors. *Adv.*

Energy Mater. **9**, 1901154 (2019).

12. Zhou, J. et al. A 6.5 nm thick anti-ferroelectric $HfAlO_x$ film for energy storage devices with a high density of 63.7 J cm^{-3}. *J. Phys. D: Appl. Phys.* **55**, 014003 (2022).

13. He, Y. et al. Superhigh energy storage density on-chip capacitors with ferroelectric $Hf_{0.5}Zr_{0.5}O_2$/antiferroelectric $Hf_{0.25}Zr_{0.75}O_2$ bilayer nanofilms fabricated by plasma-enhanced atomic layer deposition. *Nanoscale Adv.* **4**, 4648-4657 (2022).

14. Das, D., Gaddam, V. & Jeon, S. Insertion of dielectric interlayer: a new approach to enhance energy storage in $Hf_xZr_{1-x}O_2$ capacitors. *IEEE Electron Dev. Lett.* **42**, 331-334 (2021).

15. Wang, Y., Wang, Y., Zeng, H. & Wei, X. Ultra-high energy storage density of transparent capacitors based on linear dielectric ZrO_2 thin films with the thickness scaled up to hundreds nanometers. *Appl. Phys. Lett.* **120**, 023904 (2022).

16. Yi, S.-H., Lin, H.-C. & Chen, M.-J. Ultra-high energy storage density and scale-up of antiferroelectric $TiO_2/ZrO_2/TiO_2$ stacks for supercapacitors. *J. Mater. Chem. A* **9**, 9081 (2021).

17. Payne, A. et al. Dielectric, energy storage, and loss study of antiferroelectric-like Al-doped HfO_2 thin films. *Appl. Phys. Lett.* **117**, 221104 (2020).

18. Kim, K. D. et al. Scale-up and optimization of HfO_2-ZrO_2 solid solution thin films for the electrostatic supercapacitors. *Nano Energy* **39**, 390-399 (2017).

19. Silva, J. P. B. et al. Energy storage performance of ferroelectric ZrO_2 film capacitors: effect of HfO_2:Al_2O_3 dielectric insert layer. *J. Mater. Chem. A* **8**, 14171-14177 (2020).

20. Ali, F. et al. Silicon-doped hafnium oxide anti-ferroelectric thin films for energy storage. *J. Appl. Phys.* **122**, 144105 (2017).

21. Lomenzo, P. D., Chung, C.-C., Zhou, C., Jones, J. L. & Nishida, T. Doped $Hf_{0.5}Zr_{0.5}O_2$ for high efficiency integrated supercapacitors. *Appl. Phys. Lett.* **110**,

232904 (2017).

22. Park, M. H. et al. Thin $Hf_xZr_{1-x}O_2$ films: a new lead-free system for electrostatic supercapacitors with large energy storage density and robust thermal stability. *Adv. Energy Mater.* **4**, 1400610 (2014).

23. Zhang, L. et al. ALD preparation of high-*k* HfO_2 thin films with enhanced energy density and efficient electrostatic energy storage. *RSC Adv.* **7**, 8388-8393 (2017).

24. Kozodaev, M. G. et al. La-doped $Hf_{0.5}Zr_{0.5}O_2$ thin films for high-efficiency electrostatic supercapacitors. *Appl. Phys. Lett.* **113**, 123902 (2018).

25. Yang, K. et al. Energy conversion and storage using artificially induced antiferroelectricity in HfO_2/ZrO_2 nanolaminates. *Composites Part B* **236**, 109824 (2022).

26. Zhang, W. L. et al. Impact of the radiation effect on the energy storage density and wake-up behaviors of antiferroelectric-like Al-doped HfO_2 thin films. *Phys. Chem. Chem. Phys.* **22**, 21893 (2020).

27. Hoffmann, M. et al. Ferroelectric phase transitions in nanoscale HfO_2 films enable giant pyroelectric energy conversion and highly efficient supercapacitors. *Nano Energy* **18**, 154-164 (2015).

28. Payne, A. et al. Thermal stability of antiferroelectric-like Al:HfO_2 thin films with TiN or Pt electrodes. *Appl. Phys. Lett.* **120**, 232901 (2022).

29. Chen, Y. et al. Flexible $Hf_xZr_{1-x}O_2$ thin films on polyimide for energy storage with high flexibility. *IEEE Electron Dev. Lett.* **43**, 930-933 (2022).

30. Huang, R. et al. Synergistic optimization in a $0.90BaTiO_3$-$0.08Bi(Ni_{0.5}Zr_{0.5})O_3$-$0.02BiFeO_3$ thin film with high breakdown strength and energy density. *ACS Sustainable Chem. Eng.* **10**, 11041-11049 (2022).

31. Hou, C. et al. Ultrahigh energy density in $SrTiO_3$ film capacitors. *ACS Appl. Mater.*

Interfaces **9**, 20484-20490 (2017).

32. Yang, B. et al. High-entropy enhanced capacitive energy storage. *Nat. Mater.* **21**, 1074-1080 (2022).

33. Michael, E. K. & Trolier-McKinstry, S. Bismuth pyrochlore thin films for dielectric energy storage. *J. Appl. Phys.* **118**, 054101 (2015).

34. Zhu, X. et al. Remarkably enhanced energy storage properties of lead-free $Ba_{0.53}Sr_{0.47}TiO_3$ thin films capacitors by optimizing bottom electrode thickness. *J. Eur. Ceram. Soc.* **40**, 5475-5482 (2020).

35. Jiang, X. et al. Superior energy storage $BaTiO_3$-based amorphous dielectric film with polymorphic hexagonal and cubic nanostructures. *Chem. Eng. J.* **431**, 133447 (2022).

36. Diao, C. et al. Simultaneously achieved high energy storage density and efficiency in sol-gel-derived amorphous Mn-doped $SrTiO_3$ thin films. *J. Alloys Compd.* **845**, 155636 (2020).

37. Wu, M., Yu, S., Wang, X. & Li, L. Ultra-high energy storage density and ultra-wide operating temperature range in $Bi_2Zn_{2/3}Nb_{4/3}O_7$ thin film as a novel lead-free capacitor. *J. Power Sources* **497**, 229879 (2021).

38. Zhu, X. et al. Significantly enhanced energy storage density of epitaxial $Ba_{0.53}Sr_{0.47}TiO_3$ thin films by optimizing bottom electrode material. *Ceram. Int.* **46**, 13900-13906 (2020).

39. Yu, S. et al. Energy storage and dielectric properties of a novel $Bi_{1.5}MgNb_{1.5}O_7$-$Bi_2Mg_{2/3}Nb_{4/3}O_7$ thin film. *Ceram. Int.* **47**, 1238-1243 (2021).

40. Diao, C., Liu, H., Hao, H., Cao, M. & Yao, Z. Enhanced recoverable energy storage density of Mn-doped $Ba_{0.4}Sr_{0.6}TiO_3$ thin films prepared by spin-coating technique. *J. Mater. Sci.: Mater. Electron.* **29**, 5814-5819 (2018).

41. Xie, J. et al. Energy storage properties of low concentration Fe-doped barium strontium titanate thin films. *Ceram. Int.* **44**, 5867-5873 (2018).

42. Fan, Q. et al. Realization of high energy density in an ultra-wide temperature range through engineering of ferroelectric sandwich structures. *Nano Energy* **62**, 725-733 (2019).

43. Zhu, H. et al. Achieving a record-high capacitive energy density on Si with columnar nanograined ferroelectric films. *ACS Appl. Mater. Interfaces* **14**, 7805-7813 (2022).

44. Zhao, Y. et al. Achieving an ultra-high capacitive energy density in ferroelectric films consisting of superfine columnar nanograins. *Energy Storage Mater.* **39**, 81-88 (2021).

45. Zhang, T., Li, W., Zhao, Y., Yu, Y. & Fei, W. High energy storage performance of opposite double-heterojunction ferroelectricity-Insulators. *Adv. Func. Mater.* **28**, 1706211 (2018).

46. Cheng, H. et al. Demonstration of ultra-high recyclable energy densities in domain-engineered ferroelectric films. *Nat. Commun.* **8**, 1999 (2017).

47. Xu, H. et al. Microcrystalline structure modulation and energy storage properties of $BaZr_{0.25}Ti_{0.75}O_3$ thin films. *J. Alloys Compd.* **907**, 164236 (2022).

48. Xie, J. et al. Achieving ultrahigh energy storage performance in bismuth magnesium titanate film capacitors via amorphous-structure engineering. *J. Mater. Chem. C* **7**, 13632 (2019).

49. Zhang, Y. et al. Perovskite $Sr_{1-x}(Na_{0.5}Bi_{0.5})_xTi_{0.99}Mn_{0.01}O_3$ thin films with defect dipoles for high energy-storage and electrocaloric performance. *ACS Appl. Mater. Interfaces* **11**, 37947-37954 (2019).

50. Li, Z. et al. Novel $BiAlO_3$ dielectric thin films with high energy density. *Ceram. Int.*

45, 22523-22527 (2019).

51. Ye, Y. et al. Enhancing the energy storage density of $Bi_{0.5}Na_{0.5}TiO_3$ thin films by adding an amorphous alumina. *Surf. Interfaces* **33**, 102229 (2022).

52. Pan, Z. et al. Substantially improved energy storage capability of ferroelectric thin films for application in high-temperature capacitors. *J. Mater. Chem. A* **9**, 9281-9290 (2021).

53. Zhang, L. et al. Energy storage properties in $SrTiO_3$-$Bi_{3.25}La_{0.75}Ti_3O_{12}$ thin films. *J. Alloys Compd.* **799**, 66-70 (2019).

54. Yang, B. B. et al. Ultrahigh energy storage in lead-free $BiFeO_3/Bi_{3.25}La_{0.75}Ti_3O_{12}$ thin film capacitors by solution processing. *Appl. Phys. Lett.* **112**, 033904 (2018).

55. Yang, B. B. et al. Flexible ultrahigh energy storage density in lead-free heterostructure thin-film capacitors. *Appl. Phys. Lett.* **115**, 243901 (2019).

56. Yang, B. B. et al. $Bi_{3.25}La_{0.75}Ti_3O_{12}$ thin film capacitors for energy storage applications. *Appl. Phys. Lett.* **111**, 183903 (2017).

57. Diao, C. et al. Structure and electric properties of sandwich-structured $SrTiO_3/BiFeO_3$ thin films for energy storage applications. *J. Alloys Compd.* **781**, 378-384 (2019).

58. Xu, Z., Hao, X. & An, S. Dielectric properties and energy-storage performance of $(Na_{0.5}Bi_{0.5})TiO_3$-$SrTiO_3$ thick films derived from polyvinylpyrrolidone-modified chemical solution. *J. Alloys Compd.* **639**, 387-392 (2015).

59. Liu, M., Zhu, H., Zhang, Y., Xue, C. & Ouyang, J. Energy storage characteristics of $BiFeO_3/BaTiO_3$ Bi-layers integrated on Si. *Materials* **9**, 935 (2016).

60. Xie, J. et al. Performance optimization of Mg-rich bismuth-magnesium-titanium thin films for energy storage applications. *J. Eur. Ceram. Soc.* **40**, 1243-1249 (2020).

61. Nguyen, M. D., Nguyen, C. T. Q., Vu, H. N. & Rijnders, G. Experimental evidence

of breakdown strength and its effect on energy-storage performance in normal and relaxor ferroelectric films. *Curr. Appl. Phys.* **19**, 1040-1045 (2019).

62. Acharya, M. et al. Exploring the $Pb_{1-x}Sr_xHfO_3$ system and potential for high capacitive energy storage density and efficiency. *Adv. Mater.* **34**, 2105967 (2022).

63. Li, Y. et al. Ultrahigh-energy storage properties of $(PbCa)ZrO_3$ antiferroelectric thin films via constructing a pyrochlore nanocrystalline structure. *ACS Nano* **14**, 6857-6865 (2020).

64. Nguyen, M. D., Birkhölzer, Y. A., Houwman, E. P., Koster, G. & Rijnders, G. Enhancing the energy-storage density and breakdown strength in $PbZrO_3/Pb_{0.9}La_{0.1}Zr_{0.52}Ti_{0.48}O_3$-derived antiferroelectric/relaxor-ferroelectric multilayers. *Adv. Energy. Mater.* **12**, 2200517 (2022).

65. Hu, Z., Ma, B., Koritala, R. E. & Balachandran, U. Temperature-dependent energy storage properties of antiferroelectric $Pb_{0.96}La_{0.04}Zr_{0.98}Ti_{0.02}O_3$ thin films. *Appl. Phys. Lett.* **104**, 263902 (2014).

66. Lin, Z. et al. Large energy storage density, low energy loss and highly stable $(Pb_{0.97}La_{0.02})(Zr_{0.66}Sn_{0.23}Ti_{0.11})O_3$ antiferroelectric thin-film capacitors. *J. Eur. Ceram. Soc.* **38**, 3177-3181 (2018).

67. Hao, X., Wang, Y., Zhang, L., Zhang, L. & An, S. Composition-dependent dielectric and energy-storage properties of $(Pb,La)(Zr,Sn,Ti)O_3$ antiferroelectric thick films. *Appl. Phys. Lett.* **102**, 163903 (2013).

68. Huang, X.-X., Zhang, T.-F., Wang, W., Ge, P.-Z. & Tang, X.-G. Tailoring energy-storage performance in antiferroelectric $PbHfO_3$ thin films. *Mater. Design* **204**, 109666 (2021).

69. Zhao, Y., Hao, X. & Zhang, Q. Energy-storage properties and electrocaloric effect of $Pb_{(1-3x/2)}La_xZr_{0.85}Ti_{0.15}O_3$ antiferroelectric thick films. *ACS Appl. Mater. Interfaces* **6**,

11633-11639 (2014).

70. Cai, H. et al. Significantly improved energy storage properties and cycling stability in La-doped $PbZrO_3$ antiferroelectric thin films by chemical pressure tailoring. *J. Eur. Ceram. Soc.* **39**, 4761-4769 (2019).

71. Ahn, C. W., Amarsanaa, G., Won, S. S., Chae, S. A, Lee, D. S. & Kim, I. W. Antiferroelectric thin-film capacitors with high energy-storage densities, low energy losses, and fast discharge times. *ACS Appl. Mater. Interfaces* **7**, 26381-26386 (2015).

72. Tsai, M. F. et al. Antiferroelectric anisotropy of epitaxial $PbHfO_3$ films for flexible energy storage. *Adv. Funct. Mater.* **31**, 2105060 (2021).

73. Liang, Z., Ma, C., Dai, Y., Du, X. & Liu, M. Effect of mosaicity on energy storage performance of epitaxial $BaZr_{0.35}Ti_{0.65}O_3$ films. *Appl. Phys. Lett.* **118**, 162901 (2021).

74. Hu, T.-Y. et al. Realizing high energy density and efficiency simultaneously via sub-grain modification in lead-free dielectric films. *Nano Energy* **98**, 107313 (2022).

75. Kim, J. et al. Ultrahigh capacitive energy density in ion-bombarded relaxor ferroelectric films. *Science* **369**, 81-84 (2020).

76. Pan, H. et al. Ultrahigh energy storage in superparaelectric relaxor ferroelectrics. *Science* **374**, 100-104 (2021).

77. Fan, Y., Zhou, Z., Chen, Y., Huang, W. & Dong, X. A novel lead-free and high-performance barium strontium titanate-based thin film capacitor with ultrahigh energy storage density and giant power density. *J. Mater. Chem. C* **8**, 50-57 (2020).

78. Pan, H. et al. Ultrahigh–energy density lead-free dielectric films via polymorphic nanodomain design. *Science* **365**, 578-582 (2019).

79. Lv, P. et al. 4-inch ternary $BiFeO_3$-$BaTiO_3$-$SrTiO_3$ thin film capacitor with high energy storage performance. *ACS Energy Lett.* **6**, 3873-3881 (2021).

80. Sun, Z. et al. Ultrahigh energy storage performance of lead-free oxide multilayer

film capacitors via Interface engineering. *Adv. Mater.* **29**, 1604427 (2017).

81. Pan, H. et al. Giant energy density and high efficiency achieved in bismuth ferrite-based film capacitors via domain engineering. *Nat. Commun.* **9**, 1813 (2018).

82. Pan, H. et al. Enhanced electric resistivity and dielectric energy storage by vacancy defect complex. *Energy Storage Mater.* **42**, 836-844 (2021).

83. Sun, Z. et al. Modifying energy storage performances of new lead-free system ferroelectric capacitors through interfacial stress. *Appl. Surf. Sci.* **559**, 149992 (2021).

84. Pan, H. et al. $BiFeO_3$-$SrTiO_3$ thin film as a new lead-free relaxor-ferroelectric capacitor with ultrahigh energy storage performance. *J. Mater. Chem. A* **5**, 5920-5926 (2017).

85. Instan, A. A., Pavunny, S. P., Bhattarai, M. K. & Katiyar, R. S. Ultrahigh capacitive energy storage in highly oriented $Ba(Zr_xTi_{1-x})O_3$ thin films prepared by pulsed laser deposition. *Appl. Phys. Lett.* **111**, 142903 (2017).

86. Nguyen, C. T. Q., Vu, H. N. & Nguyen, M. D. High-performance energy storage and breakdown strength of low-temperature laser-deposited relaxor PLZT thin films on flexible Ti-foils. *J. Alloys Compd.* **802**, 422-429 (2019).

87. Sun, Z. et al. Large energy density, excellent thermal stability, and high cycling endurance of lead-free $BaZr_{0.2}Ti_{0.8}O_3$ film capacitors. *ACS Appl. Mater. Interfaces* **9**, 17096-17101 (2017).

88. Wang, J. et al. Optimized energy-storage performance in Mn-doped $Na_{0.5}Bi_{0.5}TiO_3$-$Sr_{0.7}Bi_{0.2}TiO_3$ lead-free dielectric thin films. *Appl. Surf. Sci.* **571**, 151274 (2022).

89. Wang, F., Chen, J., Tang, Z., Guo, F. & Zhao, S. High energy storage properties for the lead-free NBT-0.1BFO-0.068La relaxor ferroelectric film. *J. Alloys Compd.* **854**, 157306 (2021).

90. Zhang, Y. et al. Interlayer coupling to enhance the energy storage performance of

$Na_{0.5}Bi_{0.5}TiO_3$-$SrTiO_3$ multilayer films with the electric field amplifying effect. *J. Mater. Chem. A* **6**, 24550-24559 (2018).

91. Wang, C., Sun, N. & Hao, X. Dielectric property and energy-storage performance of (1-x)$PbTiO_3$-x$Bi(Mg_{0.5}Zr_{0.5})O_3$ relaxor ferroelectric thin films. *J. Mater. Sci.: Mater. Electron.* **31**, 2063-2072 (2020).

92. Nguyen, M. D., Houwman, E. P., Do, M. T. & Rijnders, G. Relaxor-ferroelectric thin film heterostructure with large imprint for high energy-storage performance at low operating voltage. *Energy Storage Mater.* **25**, 193-201 (2020).

93. Song, D. P. et al. Energy storage in $BaBi_4Ti_4O_{15}$ thin films with high efficiency. *J. Appl. Phys.* **125**, 134101 (2019).

94. Wang, F., Zhu, C. & Zhao, S. High energy storage density of NBT-0.10BFO solid solution films. *Ceram. Int.* **47**, 8653-8658 (2021).

95. Park, C.-K. et al. Nano-size grains and high density of 65PMN-35PT thick film for high energy storage capacitor. *Ceram. Int.* **44**, 20111-20114 (2018).

96. Peng, B. et al. Low-temperature-poling awakened high dielectric breakdown strength and outstanding improvement of discharge energy density of $(Pb,La)(Zr,Sn,Ti)O_3$ relaxor thin film. *Nano Energy* **77**, 105132 (2020).

97. Wang, F., Zhu, C. & Zhao, S. Good energy storage properties of $Na_{0.5}Bi_{0.5}TiO_3$ thin films. *J. Alloys Compd.* **869**, 159366 (2021).
